# Late-Onset MS: Disease Course and Safety-Efficacy of DMTS

**DOI:** 10.3389/fneur.2022.829331

**Published:** 2022-03-10

**Authors:** Maria Chiara Buscarinu, Roberta Reniè, Emanuele Morena, Carmela Romano, Gianmarco Bellucci, Antonio Marrone, Rachele Bigi, Marco Salvetti, Giovanni Ristori

**Affiliations:** ^1^Department of Neuroscience, Mental Health, and Sensory Organs, Sapienza University, Rome, Italy; ^2^Neuroimmunology Unit, IRCCS Fondazione Santa Lucia, Rome, Italy; ^3^Department of Clinical-Experimental Neuroscience and Psychiatry, Sapienza University, Rome, Italy; ^4^IRCCS Istituto Neurologico Mediterraneo (INM) Neuromed, Pozzilli, Italy

**Keywords:** late onset multiple sclerosis, immunosenescence, disease modifying therapies, efficacy, safety

## Abstract

Multiple sclerosis (MS), an inflammatory demyelinating and neurodegenerative disease of the central nervous system, usually begins between the ages of 20 and 49 years, though in rare cases it is diagnosed in childhood and adolescence before the age of 18 years, or at the age of 50 years and later. When the onset of the disease occurs at 50 years or older it is conventionally defined as late onset MS (LOMS). Compared to classical MS, the LOMS is characterized by progressive course, a greater delay in diagnosis and a higher prevalence of motor disability. The older the patients, the greater is the risk of comorbidities that can negatively influence the course of the disease and can limit therapeutic strategies. To date, there is no study focused on the efficacy of Disease Modifying Therapies (DMT) in older patients with MS. The only data available are retrievable from subgroup analysis from phase-3 trials of DMT efficacy. In this work, we discuss how the aging process influences the onset, the clinical course and the therapeutic approach in LOMS.

## Introduction

Multiple sclerosis (MS) is an inflammatory demyelinating and neurodegenerative disease of the central nervous system affecting million people worldwide (1). It is the major cause of non-traumatic neurologic disability in young adults (2). MS is usually diagnosed between the ages of 20 and 49 years, though in rare cases MS is observed in childhood and adolescence before the age of 18 years, or at the age of 50 years and later (3).

When the onset of the disease occurs at 50 years or older it is conventionally defined as late onset MS (LOMS). The prevalence rates of LOMS range from 0.6 to 12% (4, 5). The mean age at onset was between 53.8 and 67 years; the 25.3% with relapsing reminting (RR) MS progressed to secondary progressive (SP) MS (6).

Compared to classical MS, the LOMS is characterized by progressive course, a greater delay in diagnosis and a higher prevalence of motor disability. The older the patients, the greater is the risk of comorbidities that can negatively influence the course of the disease and can limit therapeutic strategies. To date, there is no study focused on the efficacy of Disease Modifying Therapies (DMT) in older patients with MS. The only data available are retrievable from subgroup analysis from phase-3 trials on the efficacy of disease-modifying therapies (DMT).

The advanced age exerts a great impact in the main aspects of the disease: the clinical course, the pathological and immunological processes, and the therapeutic choices. The aim of this review is discussing how the aging process influences the onset and the clinical course of MS, as well as to survey the issues for a therapeutic approach in LOMS. Since informative data on the relationship between age, MS and DMT are largely lacking, a pressing problem in clinical practice is to evaluate the safety of certain DMT in LOMS people. To examine this topic, we searched PubMed for all articles published from database inception to October 2021, with no language limitations. Keywords included: late onset multiple sclerosis; elderly multiple sclerosis; immunosenescence; disease modifying therapies.

## Age and MS: Immunosenescence and Disease Course

The weakening of the immune system associated with the natural aging (i.e., the immunosenescence) might be at least partly responsible for the transition of the disease course from an inflammatory to a neurodegenerative phenotype.

To evaluate age-related immunologic alterations in MS, Eschborn et al. compared immune signatures in peripheral blood and CSF by flow cytometry in patients with RR or primary progressive (PP) MS and respective controls (HD). The authors observed signs of premature immune aging in young patients with MS with alterations in immunoregulatory and costimulatory molecules that were comparable to those observed in elderly HDs. The characterization of major immune cell populations revealed an age-dependent decrease in the proportions of B cells and CD8+ T cells with a concomitant increase in CD4 T cells in HDs. In MS patients, these age-dependent alterations were significant for CD4+ and CD8+ T cells. In aged controls and patients, a decrease in naive CD8 T cells and a reciprocal increase in CD8 memory T cells was observed, especially in patients with PPMS compared with patients with RRMS. In an additional independent cohort, the same authors studied age-dependent alterations in immune cell composition and activation status in the CSF of patients with RR and PP disease as well as in non-inflammatory diseases. They observed an age-dependent decrease in counts of B and T cells, plasma cells and natural killer cells in patients with PPMS, but not in patients with RRMS, suggesting an age-dependent decrease in immune cell infiltration into the CSF of PPMS patients (7).

Numerous studies have demonstrated a decreased capacity for neurological repair with aging. Microglia and macrophages, innate immune cells important for central nervous system (CNS) regeneration, undergo senescence in distinct ways, that negatively impact the repair response in the aging CNS. Macrophages are less able to produce a functional pro-inflammatory response, while microglial cells exhibit an exaggerated proinflammatory response, a phenomenon referred to as microglia priming. Both aging microglia and macrophages exhibit deficits in phagocytic and chemotactic functions. Intervening with stimulation that may lead to a rejuvenation of aging macrophage/microglia may preserve neurological integrity and promote regeneration in the aging central nervous system (8).

There seems to be an increased prevalence of LOMS as well as of very-late-onset MS (VLOMS; conventionally the cases after 60 years). The rise in these forms is probably due to increased longevity during the last decades (9). In a recent work, whose aim was to compare demographic and clinical features of individuals with early onset, adult, and late onset MS, Mirmosayyeb et al. evidenced that individuals with LOMS have more frequently motor dysfunctions, sensory disturbances and visual impairments. The mean age at onset was 53.8 years and the disease affected more often the man (6).

Other works showed that these patients have increased risk of presenting an initial PP clinical onset, an earlier conversion to a SP disease, as well as earlier reaching of severe disability (10). In fact, studies involving patients with late onset reported a significantly higher disability: over 90% of the patients had an Expanded Disability Status Scores (EDSS) above 6.0 (11), with spinal demyelinating lesions and substantial spinal cord atrophy (12). Sixty% of cases became wheelchair-dependent or bedridden, with frequent accompanying symptoms, such as spasticity, sphincter and urinary disturbances, muscle aches (13).

In addition to motor decline, these patients more frequently present a marked cognitive impairment due to a high burden of cortical lesions, the presence of meningeal lymphoid follicle-like structures and a substantial increase in diffuse brain atrophy (14–18). Peculiarities in MRI of LOMS cases are a reduced chance of detecting active lesions and an increased possibility of detecting smoldering plaques (19), which are often also termed chronically active. They demonstrate lesion-specific rim activity associated with iron-laden macrophages and amplification of the oxidative injury owing to ferritin accumulation, being particularly associated with the onset of progressive disease and with the accelerated accumulation of physical disability (20).

The risk of comorbidities can negatively influence the course of the disease and can limit therapeutic strategies. Among those with the highest incidence, there were stroke and cancer. The five most prevalent comorbidities were depression, anxiety, hypertension, hyperlipidaemia and chronic lung disease. Thyroid disease and psoriasis were the most prevalent autoimmune diseases, while the tumors with the highest incidence in this MS population were the head-neck, breast and digestive system cancers (21).

The distinctive features of LOMS should be taken into consideration for the choice of treatment. The immunosenescence could in fact negatively influence the efficacy of Disease Modifying Therapy (DMT). On the other hand, some therapies could increase the risk of comorbidity or may be relatively contraindicated in these forms of MS.

## Age and Efficacy of DMT

Nowadays, treatment of older patients affected by MS can be really challenging. Patients with LOMS are less frequently exposed to DMTs and consequently very little is known about the efficacy of DMTs in this understudied old population. However, considering the increase of older people affected by MS, it seems clear that a better understanding of the characteristics of these patients and their potential response to DMTs is needed (22).

A recent meta-analysis confirms that age results an essential modifier of drug efficacy in patients with MS (23). Aging and immunosenescence in turn interact with the progressive phase of the disease that tend to compare as age increases. The role of progression may thus have a key role when we evaluate the efficacy of DMTs related to the age. Actually, during the fifth decade MS patients frequently experience a transition to a progressive disease with a shift from active inflammation to a compartmentalized inflammation and a faster accumulation of disability. This different inflammatory milieu in elderly raise doubts about the efficacy of immunomodulatory agents that are active against the peripheral inflammatory process underlying MS pathogenesis (9, 24).

To date there are no studies focusing on the efficacy of DMTs in RRMS in the old patients. The only available data are retrievable from subgroup analysis performed on 3-phase trials of DMT or from observational retrospective studies. All the clinical trials exclude patients older than 55 years. So, it could be useful to define the “old” patients those who are more than 40 years old.

A recent meta-analysis on clinical trials of DMTs for RRMS demonstrated efficacy in treating disease activity independent of age; nevertheless, the authors acknowledge that clinical trials select for patients with baseline disease activity, not representing real-world patients with RRMS, where disease activity declines with age (25).

However, the difference in age between clinical trials population and real-world data population is constantly growing. Clinical trial results seem to be not appropriate to define an age-dependent relationship with efficacy in general MS population. For this reason, further real-world studies are needed not only to define a clear relationship between DMT efficacy and age, but also the safety of DMT discontinuation (22).

Interferon-beta (IFN-β) represents the most frequently used drug in LOMS. Shirani et al. (22) performed a retrospective observational study in which they observed the relationship between IFN-β and disability progression in older RRMS: they found that IFN-β use was not statistically related with a slowing of disability progression (22) However, a *post hoc* analysis using data from a non-interventional, prospective cohort study of patients older than 40 years with MS and starting interferon beta-1b (IFNB-1b) treatment within 6 months before study entry (NCT00787657; BEACON: BEtaferon prospective study on Adherence, COping and Nurse support) demonstrated that these patients had benefits in using IFN-β during the observational period of 2 years (24).

Among the other first-line DMT, dimethyl fumarate (CONFIRM trial; NCT00451451) (26), peginterferon-β-1a (ADVANCE trial; NCT00906399) (27), and teriflunomide (TEMSO trial; NCT00134563) (28) reduced annual relapses rate (ARR) in both young and old patients (threshold of 38 years for teriflunomide and 40 years for the others); however, all these DMTs failed to reduce the risk of disability accumulation.

A *post hoc* analysis of teriflunomide clinical trials and their extensions, as well as real world studies demonstrated efficacy on clinical outcomes regardless of age and there was no increase in infection or death in older patients (29).

In AFFIRM (NCT00027300) and SENTINEL (NCT00030966) studies, Natalizumab failed to reduce progression in patients with MS older than 40 years; in this case factors such as male sex, EDSS score higher than 3.5 and fewer than 9 baseline T2 lesions turn out to be predictors of non-responsiveness (30). Fingolimod showed similar results in the FREEDOMS trial (NCT00289978), being not able to reduce disability progression and relapses in patients older than 40 years, compared to placebo (31).

Concerning second-line approaches, a *post hoc* analysis from the randomized CARE-MS (NCT00530348, NCT00548405) trials showed that alemtuzumab did not show different efficacy in young and old patients, evaluating both inflammatory activity and disability accumulation (32).

Among the more recent DMTs, Ocrelizumab, a B cell-depleting anti-CD20 antibody, failed to reduce ARR in patients older than 40 years (33). Ozanimod and siponimod, that are second-generation sphingosine-1-phosphate receptor modulators (selectively directed against 1,5-S1PR), showed different effects on MS measure outcomes: ozanimod failed to reduce both relapses and disability progression. (NCT02047734; NCT02294058), while siponimod resulted the first DMT that showed an efficacy to reduce disability accumulation in SPMS (NCT01665144) (34).

Disability progression represents the hardest challenge in MS management. In MS progressive forms, the neurodegeneration processes are strictly connected with the decreases of CNS capacity to remyelinate with age. In particular, the failure of oligodendrocyte precursor cells (OPCs) to differentiate into myelinating oligodendrocytes represents a major key in this process. Trial with metformin showed potential to induce maturation of OPCs and following remyelination in aged rodents (35). The cellular milieu surrounding axons, myelin sheaths and OPCs, has a key role to promote remyelination. In particular, microglia and macrophages are responsible for the phagocytosis process to eliminate myelin debris, which have a negative impact in restoring myelination and OPCs maturation. Niacin or vitamin B3, upregulating CD36 expression on microglia, promote myelin debris elimination, representing an interesting therapeutic strategy in chronic forms of MS (36). Also the senolytic drug class, such as the tyrosine kinase inhibitor dasatinib and quercetin, have been shown to potentially decrease the accumulation of senescent cells in older mice and to represent a therapeutic option for age-related pathophysiology of MS (37).

In the context of the rejuvenation research, the recovery of thymus functions may reduce the defects in negative selection and in the generation of Treg cells. An age-related IL-7 decrease is shown with a consequent thymus involution (38). Proposed treatment with IL-7 results in a higher number of recent memory CD8+ T cells growth (39). Likely, IL-22 is involved in thymopoiesis and treatment with this molecule has been shown to enhance thymic recovery (40). Finally, reduce the plethora of stimuli which led to recurrent inflammatory activations, as common infections of influenza virus and CMV, maybe another approach to reduce immunosenescence process. In this context, specific vaccination could be used to prevent persistent stimulation of the immune system, reducing the impact of peripheral inflammaging and potential triggers MS reactivation (41).

## Age and Safety of DMT

The age-related changes that take place in the immune system, a process known as immunosenescence generally result in a higher susceptibility to infections, a reduced response to vaccines and a higher prevalence of autoimmunity and neurodegenerative disorders. These processes may affect the safety of DMT, so that potentially severe adverse events are more common in elderly patients.

Data from elder MS patients are limited and of special interest in view of the fact that an increasing proportion of patients, often after long-term management of their disease, are now in the higher age groups: In 2004, an analysis of data from a large MS registry in the United States revealed that ~14% of patients with MS were ≥65 years of age (9). The clinical trials of disease-modifying therapies for RRMS were not designed to assess efficacy in aging patients. In fact, the pivotal clinical trials of the most widely used DMT specifically excluded individuals aged >45 years (glatiramer acetate), >50 years (natalizumab and alemtuzumab) and >55 years (IFNβ1a, dimethyl fumarate, fingolimod and teriflunomide). A currently recruiting phase III study of ocrelizumab in patients with progressive MS will have the highest upper age limit (65 years) used thus far in MS clinical trials.

There are two main serious adverse events caused by the long-term use of DMT in elderly: infections, such as progressive multifocal leukoencephalopathy (PML), and the potential DMT-induced cancer risk. The age-induced immunosenescence and the loss of lymphocyte functional capacity may increase the risk for occurrence of PML in MS patients treated with second-line DMTs.

The seropositivity to John-Cunningham virus (JCV), a known risk factor for PML, grows with age: an average of 10.8% conversion per year was reported (42). PML is a rare but potentially fatal complication of different DMTs. Prosperini et al. investigated if age at treatment start affects the time to onset of natalizumab-related PML. The authors showed that patients older than 50 years had a more than doubled-increased risk for an earlier PML onset (HR = 2.11, *p* = 0.006) (43). Along the same line, Jin Nakahara et al. describe 3 of the 21 registered cases of fingolimod-associated PML (without a previous natalizumab therapy) and all the patients were older than 45 years (44). In another work by Berger et al., ten out of 15 fingolimod-related PML patients were older than 50 years. In this series the patients were ~10 years older compared with those with natalizumab-associated PML (45). Higher age may also constitute a risk factor for the rare occurrence of other opportunistic infections, such as cryptococcal meningitis during fingolimod therapy.

Teriflunomide is a once-daily oral immunomodulator approved after trials with an age limit of 55 years. A non-interventional study with teriflunomide (TAURUS-MS I) included a large cohort of real-world MS patients in Germany with data derived from 1,128 patients: 558 (49.5%) patients were above 45 years old; 131 patients in the age group >55–65; and 19 patients over 65 years old. The number of patients with AEs was lowest in patients aged 26–35 years (29.2%); serious AE were 7.7% in patients aged 26–35 years and 15.0% in patients aged 46–55 years; the rate of discontinuation was higher in patients >45 years (62.9%) (46).

Changes in serum Ig levels have been reported in MS patients (47). They can be exacerbated when B-cell depleting drugs are used as DMT. In fact reduced blood concentration of IgG, IgM, and/or IgA is known to occur in patients treated with B-cell–depleting therapy (secondary antibody deficiency), including ocrelizumab. In in the Pivotal Phase III Trials of Ocrelizumab a reduction in serum Ig levels was observed, at an approximate mean rate of 3–4% per year for IgG (48). The reduced serum IgG concentrations may lead to false-negative results in JCV antibody index test in patients treated with anti-CD20 therapies (49).

**Figure 1:**
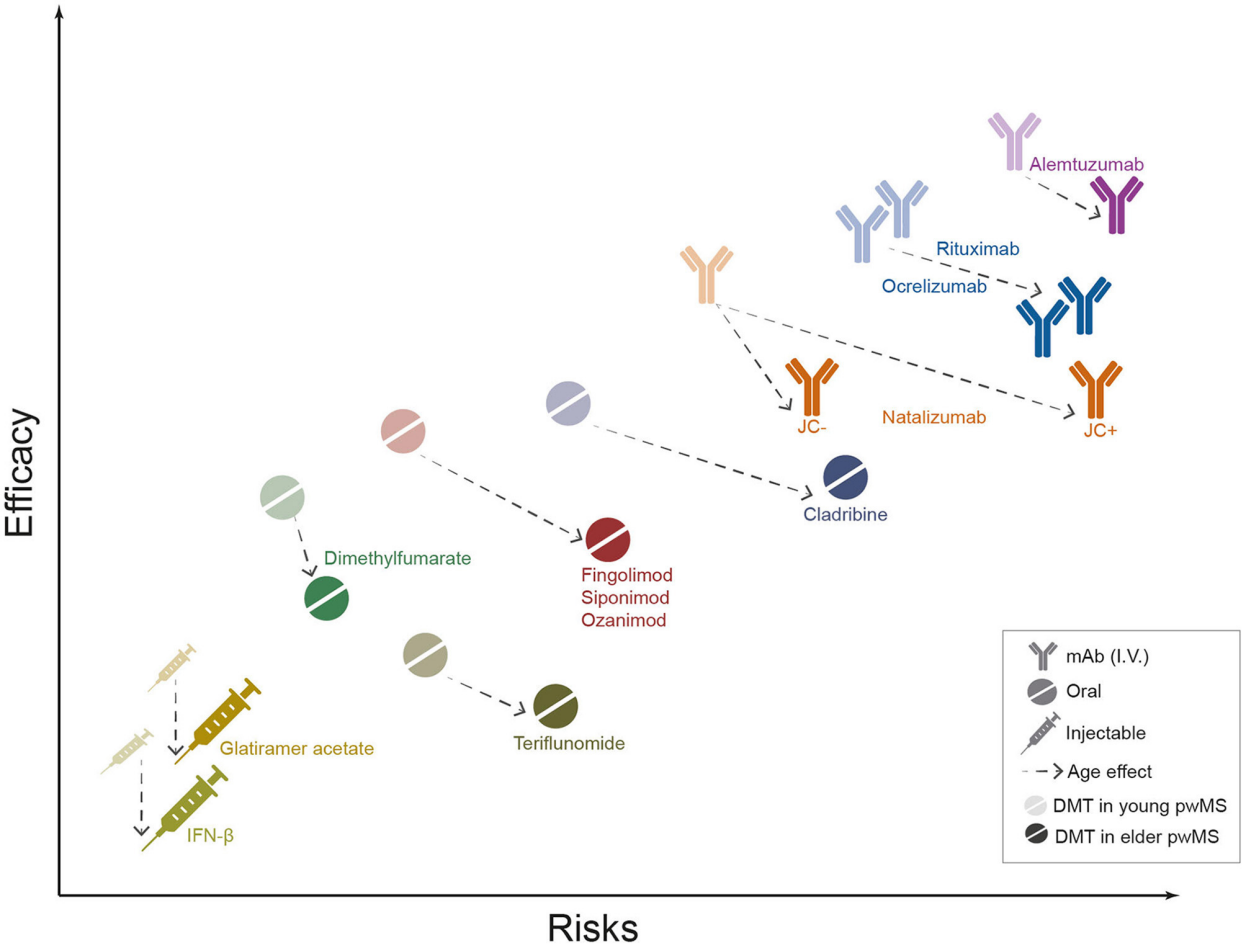
Safety and efficacy of MS DMTs in elder people with MS (pwMS). The graph shows the position of currently used MS drugs in terms of efficacy (*y axis*) and safety (expressed as growing risk burden, *x axis*). Semi-transparent icons represent DMTs effects in younger pwMS, while solid icons display the estimated effects in elderly. Dotted arrows illustrate the estimated effect of age on DMT safety and efficacy. The drugs' profiles are based on the authors' judgment, educed from the interpretation of currently available literature data. mAbs, monoclonal antibodies; I.V., intravenous administration; IFN-B, Interferon β.

Overall, these data suggest caution in DMT choice for elder patients to prevent the high risk of serious infection and the possibility of underestimating the PML risk (Figure 1).

Age above 50 in general are associated with increase in incidence rates for many types of cancer in the general population. Prosperini et al. did a meta-analysis to investigate how age could influence safety in MS patients under DMTs. They demonstrated that the interaction of age with depleting drugs (alemtuzumab, cladribine, and ocrelizumab) explained ~23% of the variance in neoplasm rate. The authors also estimated a higher neoplasm rate in patients treated with depleting agents compared to patients taking other DMTs above an average age of 45 years (50).

## Future Perspectives

Overall, informative data on the relationship between age, MS and DMT are largely lacking. Immunosenescence, nature of the DMT and age at onset of MS interact each other, challenging the possibility of designing studies aimed at disentangling the underlying mechanisms of the interplay. Real-world data and post-marketing surveillance are certainly of interest considering the fact that many patients who started DMT over the last decades are currently older than the age limits usually used in clinical trials.

A pressing problem in clinical practice is the safety of certain DMT in LOMS people: especially the depleting drugs seem to pose older subjects at higher risk of serious infections or cancer. This topic warrants further study specifically designed to quantify the risk and to disclose the better strategies to minimize such a risk.

## Author Contributions

All authors listed have made a substantial, direct, and intellectual contribution to the work and approved it for publication.

## Conflict of Interest

MS receives research support and has received fees as speaker from Sanofi, Biogen, Roche, Novartis, Bayer Schering, and Merck Serono. MB has received fees as speaker from Sanofi, Biogen, Roche, Merck Serono and Novartis. The remaining authors declare that the research was conducted in the absence of any commercial or financial relationships that could be construed as a potential conflict of interest.

## Publisher's Note

All claims expressed in this article are solely those of the authors and do not necessarily represent those of their affiliated organizations, or those of the publisher, the editors and the reviewers. Any product that may be evaluated in this article, or claim that may be made by its manufacturer, is not guaranteed or endorsed by the publisher.
